# cla-miR164-*NO APICAL MERISTEM* (*ClNAM*) regulates the inflorescence architecture development of *Chrysanthemum lavandulifolium*

**DOI:** 10.1093/hr/uhae039

**Published:** 2024-02-22

**Authors:** Junzhuo Li, Xiaohui Wen, Qiuling Zhang, Yuankai Tian, Ya Pu, Jiaying Wang, Bo Liu, Yihan Du, Silan Dai

**Affiliations:** Beijing Key Laboratory of Ornamental Plants Germplasm Innovation & Molecular Breeding, National Engineering Research Center for Floriculture, Beijing Laboratory of Urban and Rural Ecological Environment, Key Laboratory of Genetics and Breeding in Forest Trees and Ornamental Plants of Ministry of Education, Beijing Forestry University, School of Landscape Architecture, Beijing Forestry University, 35 East Qinghua Road, Beijing, 100083, China; Beijing Key Laboratory of Ornamental Plants Germplasm Innovation & Molecular Breeding, National Engineering Research Center for Floriculture, Beijing Laboratory of Urban and Rural Ecological Environment, Key Laboratory of Genetics and Breeding in Forest Trees and Ornamental Plants of Ministry of Education, Beijing Forestry University, School of Landscape Architecture, Beijing Forestry University, 35 East Qinghua Road, Beijing, 100083, China; Flower Research and Development Center, Zhejiang Academy of Agricultural Sciences, Hangzhou 311202, China; Beijing Key Laboratory of Ornamental Plants Germplasm Innovation & Molecular Breeding, National Engineering Research Center for Floriculture, Beijing Laboratory of Urban and Rural Ecological Environment, Key Laboratory of Genetics and Breeding in Forest Trees and Ornamental Plants of Ministry of Education, Beijing Forestry University, School of Landscape Architecture, Beijing Forestry University, 35 East Qinghua Road, Beijing, 100083, China; Beijing Key Laboratory of Ornamental Plants Germplasm Innovation & Molecular Breeding, National Engineering Research Center for Floriculture, Beijing Laboratory of Urban and Rural Ecological Environment, Key Laboratory of Genetics and Breeding in Forest Trees and Ornamental Plants of Ministry of Education, Beijing Forestry University, School of Landscape Architecture, Beijing Forestry University, 35 East Qinghua Road, Beijing, 100083, China; Beijing Key Laboratory of Ornamental Plants Germplasm Innovation & Molecular Breeding, National Engineering Research Center for Floriculture, Beijing Laboratory of Urban and Rural Ecological Environment, Key Laboratory of Genetics and Breeding in Forest Trees and Ornamental Plants of Ministry of Education, Beijing Forestry University, School of Landscape Architecture, Beijing Forestry University, 35 East Qinghua Road, Beijing, 100083, China; Beijing Key Laboratory of Ornamental Plants Germplasm Innovation & Molecular Breeding, National Engineering Research Center for Floriculture, Beijing Laboratory of Urban and Rural Ecological Environment, Key Laboratory of Genetics and Breeding in Forest Trees and Ornamental Plants of Ministry of Education, Beijing Forestry University, School of Landscape Architecture, Beijing Forestry University, 35 East Qinghua Road, Beijing, 100083, China; Beijing Key Laboratory of Ornamental Plants Germplasm Innovation & Molecular Breeding, National Engineering Research Center for Floriculture, Beijing Laboratory of Urban and Rural Ecological Environment, Key Laboratory of Genetics and Breeding in Forest Trees and Ornamental Plants of Ministry of Education, Beijing Forestry University, School of Landscape Architecture, Beijing Forestry University, 35 East Qinghua Road, Beijing, 100083, China; Beijing Key Laboratory of Ornamental Plants Germplasm Innovation & Molecular Breeding, National Engineering Research Center for Floriculture, Beijing Laboratory of Urban and Rural Ecological Environment, Key Laboratory of Genetics and Breeding in Forest Trees and Ornamental Plants of Ministry of Education, Beijing Forestry University, School of Landscape Architecture, Beijing Forestry University, 35 East Qinghua Road, Beijing, 100083, China; Beijing Key Laboratory of Ornamental Plants Germplasm Innovation & Molecular Breeding, National Engineering Research Center for Floriculture, Beijing Laboratory of Urban and Rural Ecological Environment, Key Laboratory of Genetics and Breeding in Forest Trees and Ornamental Plants of Ministry of Education, Beijing Forestry University, School of Landscape Architecture, Beijing Forestry University, 35 East Qinghua Road, Beijing, 100083, China

## Abstract

*Chrysanthemum* × *morifolium* has great ornamental and economic value on account of its exquisite capitulum. However, previous studies have mainly focused on the corolla morphology of the capitulum. Such an approach cannot explain the variable inflorescence architecture of the chrysanthemum. Previous research from our group has shown that *NO APICAL MERISTEM* (*ClNAM*) is likely to function as a hub gene in capitulum architecture in the early development stage. In the present study, *ClNAM* was used to investigate the function of these boundary genes in the capitulum architecture of *Chrysanthemum lavandulifolium*, a closely related species of *C.* × *morifolium* in the genus. Modification of *ClNAM* in *C. lavandulifolium* resulted in an advanced initiation of the floral primordium at the capitulum. As a result, the receptacle morphology was altered and the number of florets decreased. The ray floret corolla was shortened, but the disc floret was elongated. The number of capitula increased significantly, arranged in more densely compounded corymbose synflorescences. The yeast and luciferase reporter system revealed that *ClAP1*, *ClRCD2*, and *ClLBD18* target and activate *ClNAM*. Subsequently, *ClNAM* targets and activates *ClCUC2a*/*c*, which regulates the initiation of floral and inflorescence in *C. lavandulifolium*. *ClNAM* was also targeted and cleaved by cla-miR164 in this process. In conclusion, this study established a boundary gene regulatory network with cla-miR164-*ClNAM* as the hub. This network not only influences the architecture of capitulum, but also affects compound corymbose synflorescences of the *C*. *lavandulifolium*. These results provide new insights into the mechanisms regulating inflorescence architecture in chrysanthemum.

## Introduction

*Chrysanthemum*  × *morifolium* is famous for the variable and elegant shape of its capitulum. Its capitulum is formed by ray and disc florets, arranged according to the laws of the Fibonacci series on the receptacle. The morphology, number, direction, relative size, and stability of these two types of florets together determine the capitulum architecture. In addition, a few or more capitula constitute compound corymbose synflorescences at the top of stem and branch of some chrysanthemum varieties and wild species of *Chrysanthemum*. However, the mechanism of inflorescence architectures is obscured by the chrysanthemum’s complex genetic background. The study of the mechanism of inflorescence architectures in chrysanthemums is not only beneficial for the targeted breeding of inflorescence architecture in *C.* × *morifolium*, but also provide a reference point for related research on inflorescence architectures.

Previous studies have shown that the ABC(D)E-class genes in the MADS-box gene family and the *CYCLOIDEA 2* (*CYC2*-like) genes in the TCP gene family play an important role in the corolla formation stage of the capitulum (S8–S10). ABC(D)E-class genes are mainly involved in determining four whorls of floral organ identity decisions. Silencing of the B-class gene *PISTILLATA* (*PI*), *APETALA 3* (*AP3*), or the C-class gene *AGAMOUS* (*AG*) was found to result in loss of petal identity on the capitulum [1–3]. The *CYC2*-like gene is an important floral symmetry gene that affects the morphology of the capitulum by regulating the length and symmetry of the corolla of florets [4–9].

Studies for *Chrysanthemum lavandulifolium* and *Chrysanthemum vestitum* have shown that the floral primordium initiation stage (S5–S7) determines the number, identity, and position of florets on the capitulum, which directly affects capitulum architectures [10, 11]. Our previous research showed that boundary genes such as *NO APICAL MERISTEM* (*NAM*), *Lateral Organ Boundaries Domain 18* (*LBD18*), and *CUP*-*SHAPED COTYLEDON 2* (*CUC2*) are expressed during radial capitulum development and may be key genes for radiating capitulum architectures [12]. In Arabidopsis, *NAM*/*CUCs* and *LBD18* were found to have functions in establishing organ boundaries and are classified as boundary genes. Boundary genes are expressed at the organ initiation stage of plants to maintain normal organ morphology and function [13, 14].

The boundary genes have very strict expression domains. Ectopic expression leads to the formation of severe morphological variation in plants [15]. The *NAM* gene of Petunia is required for pattern formation in embryos and flowers [13]. When *NAM*/*CUC* was suppressed in *Fragaria* × *ananassa*, *Medicago truncatula*, and *Vigna radiata*, fusion between leaves or petals, a shift from compound leaves to simple leaves, and the phenotype of choripetalous flower to synpetalous flower was shown, suggesting that *NAM*/*CUC* plays an important role in maintaining the normal morphology of organs such as flowers and leaves [14, 16–21]. The mechanism of *NAM/CUC* has been shown to involve direct transcriptional regulation or protein interactions with ABC(D)E class genes and TCP genes [16, 22]. In addition, some members of the NAC gene family are targeted and cleaved by miRNA164. This mechanism is involved in organ initiation and morphological maintenance in several species [14, 18–21, 23, 24]. Many studies have suggested that the miRNA164-*NAM/CUC* module is essential for the maintenance of normal inflorescence morphology in plants and forms a regulatory network with floral development genes, such as ABC(D)E class genes and TCP genes, to regulate flower morphology. However, the function of the miRNA164-*NAM/CUC* module in complex inflorescence structures such as the capitulum has not been reported.

Although boundary genes may play an important role in the floral primordium initiation stage of capitulum development, boundary genes’ function has been poorly studied in Asteraceae plants. In this study, the biological characteristics of *NAM*-like (*ClNAM*) in *C. lavandulifolium,* which is a wild species in the genus closely related to *C.* × *morifolium*, were identified. Furthermore, the *ClNAM* overexpressing transgenic lines were constructed through the stable genetic transformation of *C. lavandulifolium*, we discovered highly specific phenotypes in those transgenic lines. Overexpression of *ClNAM* not only affected the architectures of primary inflorescence, but also secondary inflorescence (a combination of multiple primary inflorescence) of the *C. lavandulifolium*. In the capitulum (primary inflorescence), the receptacle morphology was altered and the number of florets decreased. In addition, the corolla of the ray floret was shortened, but the disc floret was elongated. In the compound corymbose synflorescences (secondary inflorescence), the number of capitulum increased significantly. This result indicates that *ClNAM* affects the inflorescence architectures of *C. lavandulifolium* by regulating the initiation of floral and inflorescence primordia. To analyse the mechanism of action of *ClNAM*, we examined the up- and down-stream genes of *ClNAM* using the yeast reporter system and the luciferase reporter system. The results showed that *ClNAM* responds to the transcriptional activation of the A-class gene *APETALA* 1 (*ClAP1*), the E-class genes *C. lavandulifolium REGULATOR OF CAPITULUM DEVELOPMENT 2* (*ClRCD2*) and the boundary gene *ClLBD18*, and is also targeted and cleaved by cla-miR164. Subsequently, *ClNAM* directly activates downstream boundary genes *ClCUC2a*/*c* expression, controlling the floral and inflorescence primordia initiation. Our results suggest a boundary gene regulatory network with cla-miR164-*ClNAM* as the hub and provide new insights into regulation of chrysanthemum inflorescence architecture.

## Results

### The biological characteristics of *ClNAM*

**Figure 1:**
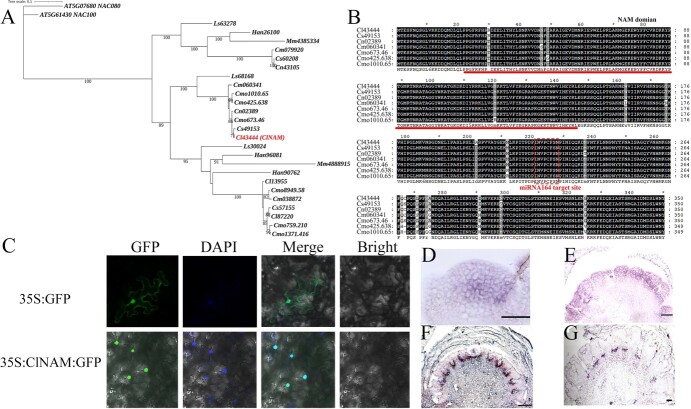
Phylogenetic position and spatiotemporal expression characteristics of *ClNAM*. (**A**) Phylogenetic position of ClNAM; the ClNAM (Cl43444) is the sequence used in this study. (**B**) Protein sequences of NAM are conserved in Chrysanthemum species. (**C**) Results of subcellular localization of ClNAM. (**D**–**G**) Expression patterns of *ClNAM* in the doming stage (S1), early stage of floret primordia initiation (S6), early stage of corolla primordia formation (S8), and late stage of corolla primordia formation (S10). bar = 200 μm. At: *Arabidopsis thaliana*; Cl: *Chrysanthemum lavandulifolium*; Cm: *Chrysanthemum makinoi*; Cmo: *Chrysanthemum* × *morifolium*; Cn: *Chrysanthemum nankingense*; Cs: *Chrysanthemum seticuspe*; Han: *Helianthus annuus*; Mm: *Mikania micrantha*; Ls: *Lactuca sativa*.

Phylogenetic analysis revealed that ClNAM (Cl43444) is a member of the NAM clade in the NAC gene family (Fig. S1A, see online supplementary material). One ortholog of ClNAM was present in diploid species of *Chrysanthemum* (*Chrysanthemum seticuspe*, *Chrysanthemum nankingense*, and *Chrysanthemum makinoi*), while three copies were conserved in the hexaploid species *C.* × *morifolium* (Fig. 1A). Multiple sequence alignment revealed that the sequences of *NAM* were conserved among *Chrysanthemum* species and all retained the cla-miR164 target binding site (Fig. 1B; Fig. S1B, see online supplementary material). The subcellular localization results showed that the ClNAM-GFP fusion protein had distinct green fluorescence only in the nucleus, indicating that the ClNAM protein was localized in the nucleus with a typical t character of transcription factors (Fig. 1C).

To understand the role of *ClNAM* in the architectures of *C. lavandulifolium* capitulum, the spatiotemporal expression characteristics of *ClNAM* during capitulum development were observed by *in situ* hybridization (Fig. 1D–G). The results showed that *ClNAM* was widely expressed in the inflorescence meristem at the doming stage (S1, Fig. 1D). At the early stage of floret primordia initiation, *ClNAM* was significantly expressed in disc floret primordia (S6, Fig. 1E). At the early stage of corolla primordia formation, the *ClNAM* expression was very strong between floret primordia, especially between disc floret primordia (S8, Fig. 1F). At the late stage of corolla primordia formation, the *ClNAM* expression gradually became weaker between florets (S10, Fig. 1G).

These results tentatively suggest that *ClNAM* has an expression pattern consistent with the boundary genes. It may be involved as a transcription factor in the initiation of floret primordia, especially of the disc floret, from the inflorescence meristem on the *C. lavandulifolium* capitulum. Because the sequence and copy number of NAM is highly conserved among *Chrysanthemum* species, this function of regulating inflorescence differentiation may also be conserved in chrysanthemums.

### The effect of overexpression *ClNAM* on the inflorescence morphology of *C. lavandulifolium*

**Figure 2:**
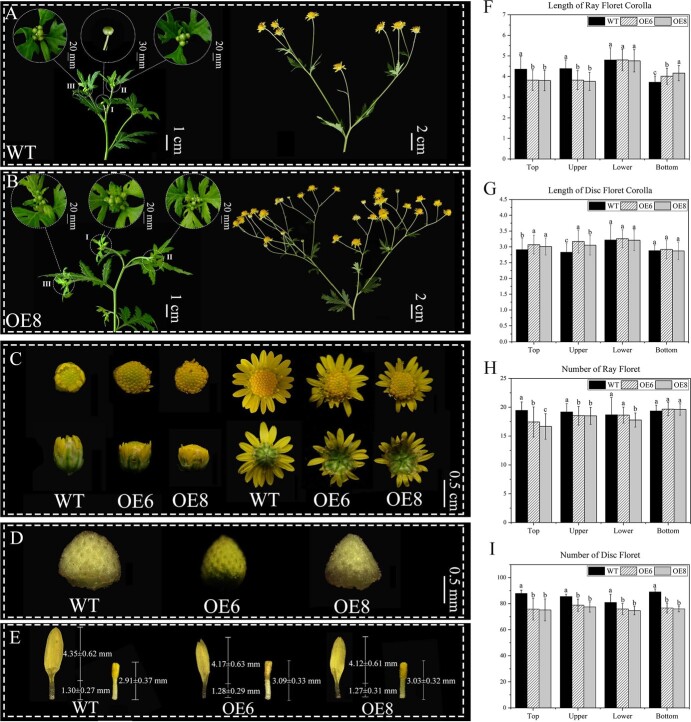
Effect of overexpression of *ClNAM* on the morphology of *Chrysanthemum lavandulifolium* inflorescences. (A–B) Overexpression of *ClNAM* caused a significant increase in the number of capitulum on top of plants. (C) Overexpression of *ClNAM* resulted in developmental delayed ray floret corolla and elongated disc corolla. (D) Overexpression of *ClNAM* caused changes in the receptacle morphology of the *C. lavandulifolium* and a decrease in the area of receptacle. (E) Overexpression of *ClNAM* resulted in shortened ray floret corolla and elongated disc corolla. (F–G) Overexpression of *ClNAM* caused a change in the length of both types of florets with the position. (H–I) Overexpression of *ClNAM* caused a change in the number of both types of florets with the position height. Note: All phenotypic data were obtained from five independent plants. The lowercase letters represent the significance of differences between the three lines; where the letters are different, there is a significant difference (*P* < 0.05).

To verify the effect of *ClNAM* on the inflorescence morphology of *C. lavandulifolium*, we constructed the transgenic lines of *35S*::*ClNAM* and made detailed observations of their phenotypic changes. Interestingly, the extent of changes in inflorescence morphology was not uniform throughout the plants. At the top of plants, WT lines plants produced 1–6 capitulum per compound corymbose synflorescence (Fig. 2A), whereas OE-*ClNAM* lines produced 4–16 per compound corymbose synflorescence (Fig. 2B). The number of capitula in the OE-*ClNAM* lines increased with the height of the branch (Fig. S2A, see online supplementary material), and the number of capitula on the bottom branches was lower than in the WT plants (Fig. S2B, see online supplementary material). This secondary branching pattern significantly altered the inflorescence morphology of *C. lavandulifolium*, resulting in the compound corymbose synflorescences also in the upper branches of the plant (Fig. S2A, see online supplementary material). Because boundary genes can also affect root growth [24, 27], we also observed root morphology in the OE-*ClNAM* lines but did not observe any significant changes (Fig. S3, see online supplementary material).

Overexpression of *ClNAM* also affected the morphology of the capitulum, with the OE-*ClNAM* lines having a significantly smaller capitulum than the WT lines (Fig. 2C). The development of ray florets was significantly suppressed in the OE-*ClNAM* lines, while the ray florets were still enveloped by the involucre in the first round of disc florets flowering. In addition, the state of ray florets development was not uniform (Fig. 2C). The corolla length of the disc florets showed the opposite trend, being longer in the OE-*ClNAM* lines than the WT lines (Fig. 2E). The extent of variation in corolla length for both types of florets was also correlated with the height of the inflorescence position and was most pronounced at the top of the plant (Fig. 2F–G).

In addition, we observed a change in receptacle morphology in the OE-*ClNAM* lines, from hemispherical to conical in the WT lines, with a change in surface area (Fig. 2D). The receptacle is the inflorescence axis of the capitulum, and all florets are closely arranged on the receptacle. The surface area of the receptacle is therefore closely related to the number of florets. The changes in the number of florets of the two types were counted separately and the numbers were found to be generally reduced in the OE-*ClNAM* lines (Fig. 2H–I). The number of ray florets was highly significantly reduced in the top of the OE-*ClNAM* lines, and gradually returned to the WT level as height decreased (Fig. 2H). In contrast, the number of disc florets on the capitulum of the OE-*ClNAM* lines was highly significantly reduced at all positions (Fig. 2I).

These results suggest that *ClNAM* influences the capitulum morphology through the number of florets on the capitulum and the length of the corolla. In addition, *ClNAM* also influenced the number of capitulum initiations on branches and had a regulatory effect on the architecture of compound corymbose synflorescences. These phenotypic changes followed a specific pattern, with the greatest degree of variation at the top of the plant and almost no difference from the wild type at the bottom. The causes of this pattern of phenotypic variation need to be further analysed.

### *ClNAM* is targeted and cleaved by cla-miR164

**Figure 3:**
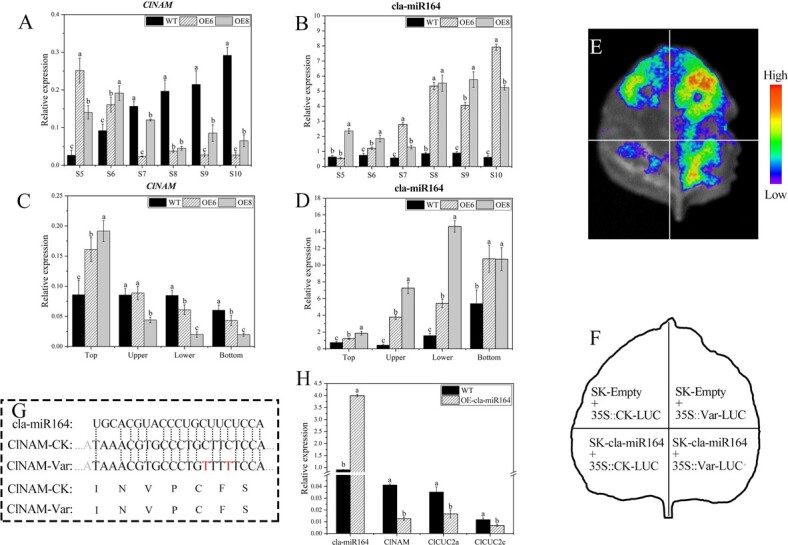
Involvement of cla-miR164 in inflorescence architecture development through targeted cleavage of *ClNAM* in *Chrysanthemum lavandulifolium*. (A–B) Expression changes of *ClNAM* and cla-miR164 during the capitulum developing from the top of *C. lavandulifolium*. (C–D) Expression changes of *ClNAM* and cla-miR164 in S6 reproductive buds of different heights in *C. lavandulifolium*. (E–F) Targeted cleave ability of cla-miR164 on *ClNAM*. (G) The targeting sites of *ClNAM* and sequences of cla-miR164 in *C. lavandulifolium*, CK: Control Check, Var: Variant. (H) Expression changes of cla-miR164, *ClNAM*, *ClCUC2a/c* in leaves of momentary overexpression cla-miR164. Note: The lowercase letters represent the significance of differences between lines; where the letters are different, there is a significant difference (*P* < 0.05).

Because members of the *NAM/CUC* subfamily are usually regulated post-transcriptionally by miRNA164 [14, 18–20, 23, 24], it was hypothesized that the specific pattern of phenotypic changes in the OE-*ClNAM* lines was related to the expression levels of *ClNAM* and cla-miR164. First, we examined the expression patterns of *ClNAM* and cla-miR164 during the capitulum development stage from the top of the plants by qRT-PCR. The results showed that *ClNAM* was consistently up-regulated with the development of the capitulum in the WT lines. In the OE-*ClNAM* lines, the expression peak of *ClNAM* was advanced to the floral primordium initiation stage (S5–S7), and instead was much lower than that of the WT lines at the corolla formation stage (S8–S10) (Fig. 3A). While cla-miR164 showed no significant expression changes during capitulum development in the WT lines, cla-miR164 showed highly significant up-regulated expression at all stages and peaked at the corolla formation stage in the OE-*ClNAM* lines (Fig. 3B). During capitulum development of the OE-*ClNAM* lines, *ClNAM* and cla-miR164 showed opposite expression trends, tentatively suggesting that *ClNAM* might be negatively regulated by cla-miR164.

The expressions of *ClNAM* and cla-miR164 were examined in reproductive buds at the S6 stage on four heights of *C. lavandulifolium*. The expression of *ClNAM* remained essentially the same in the reproductive buds of the WT lines at different heights and only slightly decreased at the bottom, while the expression of *ClNAM* in the OE-*ClNAM* lines was only significantly higher in the reproductive buds from the top than in the wild type. In addition, the expression of *ClNAM* decreased as the height of the reproductive buds decreased (Fig. 3C). In contrast, the expression pattern of cla-miR164 was the opposite, with lower expression at the top of the plant and higher expression in the lower lateral branches and bottom branches. In addition, overexpression of *ClNAM* significantly increased the expression of cla-miR164 (Fig. 3D).

Possible cla-miR164 target binding sites in *ClNAM* were projected and ligated into pGreenII 0800-LUC containing 35S promoter and co-injected with pGreenII 62-SK with cla-miR164 precursor sequence attached in the leaves of tobacco. The change in fluorescence value was then observed. The results showed that cla-miR164 had a direct targeting and cleaving ability on *ClNAM*. This targeting and cleaving ability was eliminated after 2-point mutations with synonymous substitutions in the targeting site of *ClNAM* (Fig. 3E–G). To further determine the targeted degradation of ClNAM by cla-miR164 in *C. lavandulifolium*, the *Agrobacterium* GV3101 which contains blank and 35::cla-miR164 pGreenII 62-SK plasmids was used and injected into *C. lavandulifolium* leaves and the expression of cla-miR164 and downstream target genes was measured in the treated leaves (Fig. 3H). The results showed that the expression of *ClNAM* was significantly reduced after overexpression of cla-miR164 in the leaves of *C. lavandulifolium*. In previous reports, *CUC2* is a conserved target gene of cla-miR164, and we similarly observed that *ClCUC2a/c* expression was significantly downregulated in leaves of OE-cla-miR164.

The function of cla-miR164 in *C. lavandulifolium* is to target and cleave the *ClNAM*. The expression patterns of *ClNAM* and cla-miR164 during capitulum development and in reproductive shoots of different heights indicate that overexpression of *ClNAM* leads to enriched cla-miR164 levels and there are differences between top and bottom inflorescences. Eventually, this causes a specific pattern of phenotypic variation on the OE-*ClNAM* lines. Therefore, we also determined that *ClNAM* in OE-ClNAM is not completely overexpressed, but presents a pattern of modified expression.

### The transcriptional regulation of *ClNAM* in relation to genes related to flower development

To investigate the molecular mechanism of *ClNAM* regulation of inflorescence architectures in *C. lavandulifolium*, we selected reproductive buds at the S6 stage from the top of the plants, where *ClNAM* expression was highest and phenotypic changes in inflorescence were most significant, for the construction of transcriptome libraries (Table S1, see online supplementary material). A total of 2566 differentially expressed genes were screened in the OE-*ClNAM* lines, of which 556 genes were up-regulated and 2010 genes were down-regulated (Fig. S4, see online supplementary material). Further screening of genes related to inflorescence development in the data set revealed that *ClNAM*, *ClCUC2a*, and *ClCUC2c* were up-regulated among the boundary genes in the OE-*ClNAM* lines. In addition, many ABCE-class genes of the MADS-box gene family, auxin response factors, and auxin transporter proteins were up-regulated in the OE-*ClNAM* lines (Fig. S5, see online supplementary material).

**Figure 4:**
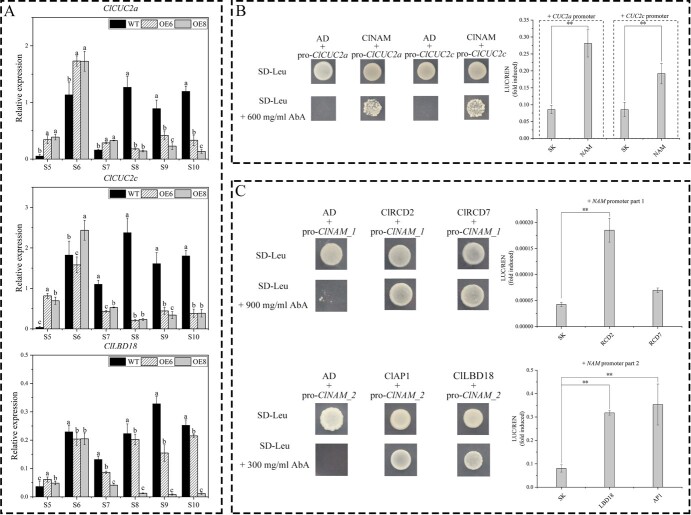
Up- and down-stream transcription factor screening of *ClNAM*. (**A**) Expression changes of boundary genes *ClLBD18*, *ClCUC2a,* and *ClCUC2c* in the OE-*ClNAM* lines. (**B**) *ClNAM* has transcriptional activation ability for *ClCUC2a* and *ClCUC2c*. (**C**) The expression of *ClNAM* is significantly activated by *ClLBD18*, the A-class gene *ClAP1,* and the E-class gene *ClRCD2*. Note: **P < 0.01. The lowercase letters represent the significance of differences between the three lines; where the letters are different, there is a significant difference (*P* < 0.05).

In a previous study, we found that *ClLBD18*, *ClNAM*, *ClCUC2a*, and *ClCUC2c* show co-expression patterns during capitulum development and may have a direct upstream-downstream relationship [12]. We examined the expression of *ClLBD18*, *ClCUC2a*, and *ClCUC2c* after the modification of *ClNAM*. The results showed that *ClCUC2a* and *ClCUC2c* had a consistent expression pattern with *ClNAM*, with the expression peak being advanced to the middle of floral primordium initiation stage (S6), while the expression at the corolla formation stage (S8–S10) was repressed, possibly by downstream genes directly regulated by *ClNAM*. In contrast, *ClLBD18* expression in the OE6 and OE8 lines varied widely and may not be a downstream gene regulated by *ClNAM* (Fig. 4A). Analysis of the promoter sequences of *ClCUC2a* and *ClCUC2c* revealed the presence of *ClNAM* binding sites on both of their promoter sequences (Fig. S6A, see online supplementary material). The results of Y1H experiments also indicated that *ClNAM* was able to bind to these regions (Fig. 4B). A dual-luciferase assay revealed the highly significant transcriptional activation ability of *ClNAM* for *ClCUC2a* and *ClCUC2c* (Fig. 4B).

Further analysis revealed there were two MADS-box genes and a conserved binding site for *LBD18* in the promoter sequence of *ClNAM* (Fig. S6A, see online supplementary material). Y1H experiments revealed that the E-class genes *ClRCD2* and *ClRCD7* were able to bind in part 1, and *ClLBD18* and the A-class gene *ClAP1* were able to bind in part 2 (Fig. 4C). The results of the dual luciferase assay showed that *ClLBD18*, *ClAP1*, and *ClRCD2* all activated the expression of *ClNAM* significantly.

These results suggest that the expression of *ClNAM* is regulated by the MADS-box gene and the boundary gene *ClLBD18*. Then, the *ClNAM* activates the downstream *ClCUC2a* and *ClCUC2c*, forming a boundary gene regulatory network with *ClNAM* as the central core. Combined with the phenotypic changes in the OE-*ClNAM* lines, we suggest that this gene regulatory network is involved in the initiation of floret and flower primordia on the capitulum and compound corymbose synflorescence of *C. lavandulifolium*, regulating the number of florets per capitulum and capitulum of compound corymbose, which in turn affects the construction inflorescence morphology of *C. lavandulifolium*.

### *ClNAM* indirectly regulates the expression of ABCE-class genes

Up-regulated expression of many MADS-box genes was observed in the transcriptome data of the OE-*ClNAM* lines. These genes are highly correlated with corolla development [25] and may be responsible for the variation in corolla length of florets in the OE-*ClNAM* lines. In a previous study, eight MADS-box genes differentially expressed in the corollas of ray and disc florets of *C. lavandulifolium* were identified, among which the B-class gene *ClAP3* and *ClPIa*, the E-class gene *ClRCD1/3/4/7* were highly expressed in the ray florets. The A-class gene *ClAP1* and the C-class gene *ClAG1* were highly expressed in the corollas of disc florets [26].

**Figure 5:**
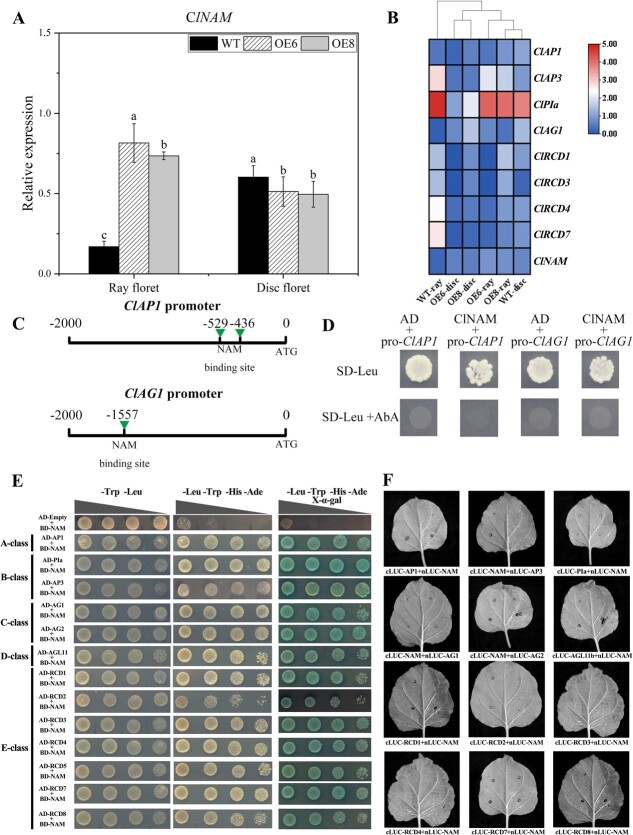
Modified *ClNAM* affects the expression of MADS-box genes that control corolla development in two types of florets. (**A**) Expression of *ClNAM* in the corollas of both types of florets after modified of *ClNAM*. (**B**) Changes in expression of key MADS-box genes in the corollas of both types of florets after modified of *ClNAM*. (**C**–**D**) *ClNAM* fails to bind to *ClAP1* and *ClAG1* promoter elements. (**E**–**F**) ClNAM was detected to widely interact with ABC(D)E-class protein in *Chrysanthemum lavandulifolium* in Y2H system, but no positive results were observed in the luciferase complementation assay. Note: The lowercase letters represent the significance of differences between the three lines; where the letters are different, there is a significant difference (*P* < 0.05).

The expression of *ClNAM* and the above-mentioned MADS-box genes were examined in the corollas of WT and OE-*ClNAM* lines. The expression of *ClNAM* was significantly increased in ray floret corollas and significantly decreased in disc floret corollas in the OE-*ClNAM* lines (Fig. 5A). In addition, the expression of *ClRCD4* and *ClRCD7* were up-regulated, and the expression of *ClAP3*, *ClPIa*, *ClAP1,* and *ClAG1* were down-regulated in the ray floret corollas of OE-*ClNAM* lines. The overall gene expression pattern was close to that of the disc floret corollas in the WT lines (Fig. 5B). These results suggest that *ClNAM* may be a key gene in maintaining the morphological development of disc corollas. When *ClNAM* was ectopically expressed in ray corollas, the ray corollas were shortened, and development was delayed, and the expression patterns of the associated MADS-box were all similar to those of disc corollas of the WT lines.

By analysing the promoter sequences of *ClRCD1*, *ClRCD4*, *ClAP3*, *ClPIa*, *ClAP1,* and *ClAG1*, we found possible binding sites of *ClNAM* in the promoter sequences of *ClAP1* and *ClAG1*. However, the results of the Y1H assay showed that ClNAM did not bind to these sites (Fig. 5C and D). This suggests that ClNAM does not directly regulate the expression of these MADS-box genes. There may be some unknown transcription factors between the *ClNAM* and MADS-box genes. In addition, we found that ClNAM widely interacts with ABC(D)E-class protein in *C. lavandulifolium* by Y2H assay (Fig. 5E), but no corresponding positive resulted from further luciferase complementation assay (LCA) and bimolecular fluorescence complementation (BiFC) (Fig. 5F; Fig. S7, see online supplementary material).

These results suggest that the change in corolla length of florets in the OE-*ClNAM* lines result from the ectopic expression of *ClNAM* followed by altered expression of related ABC(D)E-class genes. Although they are correlated in expression patterns, there is no evidence of a direct transcriptional regulatory relationship between *ClNAM* and these ABC(D)E-class genes. Their protein-level interactions can only be detected in the Y2H system, with the possibility of false positives.

## Discussion

**Figure 6:**
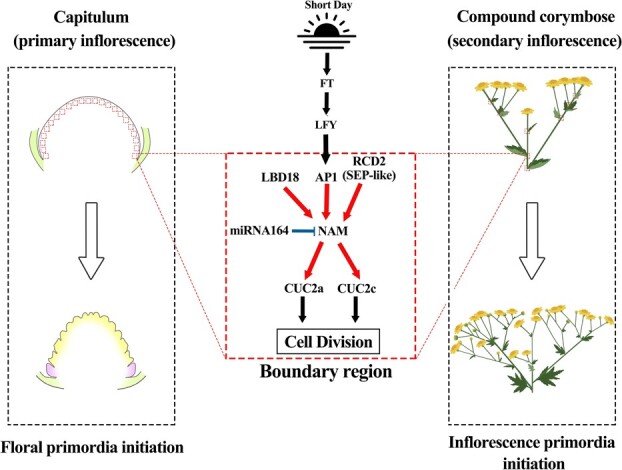
The boundary gene regulatory network with cla-miR164-*ClNAM* as the core is involved in the inflorescence architectures of *Chrysanthemum lavandulifolium.* Note: The black arrows indicate the previous findings, while the red arrows indicate the original results in this study. The blue line segment indicates the targeted degradation effect of cla-miR164 on *ClNAM*. The red boxes indicate the boundary region on the inflorescence meristem.

Predicted targets of miR164 include mRNAs encoding five members of the NAC domain transcription factor family in Arabidopsis [27]. Of these, *NAC1* is involved in the transduction of auxin signals for lateral root development, and *CUC1/CUC2* are involved in meristem development and aerial organ separation, whereas the functions of *At5g07680* and *At5g61430* have not yet been defined [28]. In this study, we found the potential function of *ClNAM*, which is the orthologous gene of *At5g07680* and *At5g6143*. Further study presents a boundary gene regulatory network with cla-miR164-*ClNAM* as the core. The results showed that *ClNAM* was activated by the *ClAP1* and *ClRCD2* as well as the boundary gene *ClLBD18*. Activating the expression of *ClCUC2a*/*c*, which induced the initiation of floret and inflorescence primordia on the inflorescence meristem, regulated the differentiation of the two types of florets and the capitula in *C. lavandulifolium*. This network influenced the establishment of primary and secondary inflorescence morphology in *C. lavandulifolium* in terms of the number of flowers (Fig. 6). This finding provides new insights into the early inflorescence development process of chrysanthemums and suggests a new pathway in the molecular regulatory network of regulation of chrysanthemums inflorescence architecture.

### cla-miR164-*ClNAM* module regulates the initiation of *C. lavandulifolium* floret and flower primordia

In previous studies of Arabidopsis, boundary genes such as *LBD18* and *CUCs* through the regulation auxin concentration and KNOX expression to inhibit the cell division, but there is no direct regulative relationship between them [14]. In the present study, we found that *ClNAM* is located between *ClLBD18* and *ClCUC2s* in the regulatory network and transmits transcriptional regulatory signals to the boundary genes. Because the capitulum structure is more elaborate in Asteraceae than in many other plants, we propose that this hierarchical transmission of regulatory signals from boundary genes is crucial for the maintenance of this complex inflorescence morphology.

Interestingly, the trends in the number of flowers in the capitulum (primary inflorescence) and in the compound corymbose synflorescence (secondary inflorescence) were different in the OE-*ClNAM* lines. The number of both types of florets in the capitulum was significantly reduced in the OE-*ClNAM* lines, and the number of disc florets in the disc center was particularly reduced. In contrast, the number of capitula increased significantly in the compound corymbose synflorescence of the OE-*ClNAM* lines. This contrast is mainly due to the different patterns of development in the two types of inflorescences. Capitula have a specific developmental pattern in which the highly compressed inflorescence axis (receptacle) is in dynamic equilibrium with the initiation of the floral primordium [10]. When the expression peak of *ClNAM* is advanced in the OE-*ClNAM* lines, the receptacle completes all florets before it is fully developed. As a result, the morphology of the receptacle is altered and the surface area is reduced, leading to a decrease in the number of florets. In the OE-*ClNAM* lines, the disc florets in the center of the disc were more affected than the ray florets at the margins, indicating that *ClNAM* regulates the initiation of disc flower primordia during receptacle development. In contrast, in the compound corymbose synflorescence of *C. lavandulifolium*, the capitulum developed gradually from the top to bottom when the inflorescence axis was fully developed, and *ClNAM* directly promoted the initiation of the capitulum primordia.

This study also revealed the *ClNAM* was targeted and cleaved by cla-miR164 in *C. lavandulifolium*. The cla-miR164 was up-regulated when in ectopic expression and overexpression of *ClNAM* and removes excess *ClNAM* to maintain normal inflorescence morphology. This mechanism led to inconsistent results in the extent of inflorescence morphological variation of the OE-*ClNAM* lines. In *Zea mays*, *Oryza sativa*, *Arabidopsis thaliana*, and *M. truncatula*, miRNA164 exhibited targeted cleavage effects on NAC gene family members to maintain normal organ morphology and function, suggesting that such regulatory effects are conserved across species [19–21, 24]. It has been shown that miRNA164 acts in conjunction with *NAM/CUC* as a homeostatic mechanism to regulate auxin signaling [27, 29, 30], while the localized enrichment of auxin is essential for floret patterning in Asteraceae [31]. In addition, auxin also has a regulatory role in the corolla morphology of ray floret in chrysanthemums [32, 33]. In the present study, many differential expressions of auxin transporter proteins and auxin response factors were found in the OE-*ClNAM* lines, suggesting that cla-miR164-*ClNAM* may affect inflorescence architectures in chrysanthemums by regulating auxin signaling (Fig. S5, see online supplementary material).

In conclusion, our results suggest that the cla-miR164-*ClNAM* module facilitates the initiation of floral and inflorescence primordium, and maintains a dynamic balance between the development of inflorescence meristem and the initiation of floral primordium in the capitulum. This function both affected the architectures of capitulum and the compound corymbose synflorescence (Fig. 6).

### *ClNAM* has a complex regulatory relationship with ABC(D)E class genes

*AP1* is not only the A-class gene that specifies sepal and petal identity but also an important floral meristem (FM) maintenance gene, regulated by the upstream *LEAFY* (*LFY*) gene activating floral meristem initiation [34–37]. *SEPALLATA* (*SEP*) in Arabidopsis is highly redundant in its function to determine floral organ identity, but *RCD*, the ortholog of the *SEP* in plants of the Asteraceae family, undergoes significant expansion and functional divergence. *RCD2/3/4/7* are not only involved in organizing the inflorescence meristem at the capitulum, but also in the orderly differentiation of the inflorescence meristem (IM) and floral meristem at the capitulum [38]. In this study, we found that *ClNAM* is directly activated by ClAP1 and ClRCD2, suggesting that the boundary genes are located downstream of the floral induction network and initiate the floral meristem after receiving upstream floral induction signals.

*In situ* hybridization showed that *ClNAM* was expressed predominantly in disc florets during the corolla formation stage. qRT-PCR results also indicated that *ClNAM* was expressed significantly more in disc corollas than in ray corollas, suggesting that *ClNAM* may also have a function in maintaining morphological differences between the corollas of the two types of florets (Fig. 5A). Although many ABC(D)E-class genes were found to be differentially expressed in the transcriptome data and in qRT-PCR experiments in the OE-*ClNAM* lines, which were accompanied by significant changes in corolla length, no evidence was found for a direct association of *ClNAM* with these ABC(D)E-class genes. Although *ClNAM* showed wide protein interactions with ABC(D)E-class genes in the Y2H assay, no corresponding positive results were observed in the luciferase complementation assay. A similar decrease in expression of all ABC(D)E-class genes with *MtNAM* was observed in the *nam* mutant from *M. truncatula*, but the regulatory relationship between the two was not elucidated [16]. These results suggest that there is a close relationship between *NAM* and these floral organ identity-determining genes, but no evidence has shown any directly targeted and regulated during floral organs development. We hypothesize that there are still unknown hub genes between *ClNAM* and ABC(D)E-class genes, or that *ClNAM* affects floral organ development through other regulatory pathways that are not causally related to changes in the expression of ABC(D)E-class genes.

## Materials and methods

### Plant materials and genetic transformation

The G1 line of *C. lavandulifolium* used in the present study was the same as *C. lavandulifolium* genome sequencing [12]. The full-length CDS (coding sequence) of *ClNAM* was cloned into the binary vector pBI121 and used to transform *Agrobacterium* GV3101. The transgenic *C. lavandulifolium* plants were obtained by the leaf disc transformation. The MS medium containing 0.1 mg 6-BA +2.0 mg/L NAA + 400 mg/L Carb was used for inducing the transformation. The MS medium containing 7 mg/L Kan + 400 mg/L Carb was used for selection. Positive plants were detected by PCR using primers 35S (F: 5′-GACGCACAATCCCACTATCC-3′) and NOS (R: 5′-AATCATCGCAAGACCGGC-3′).

### Growth conditions and phenotypic observations

These materials were planted with peat: vermiculite = 1:1. The temperature was 22 ± 1°C and the light intensity was 4000 lx. The nutritive growth was carried out under long daylight (16 h light/8 h dark). After they grew 14 leaves, they were transferred to short daylight (12 h light/12 h dark) for reproductive growth. Phenotypic observations and statistics were made at flowering. Five biological replicates were used to confirm the reliability of the observations.

### Phylogenetic analysis

The NAC protein sequences of Arabidopsis, which are used as a reference sequence for BLAST were downloaded from the Plant Transcription Factor Database (http://planttfdb.gao-lab) [39]. Genomic data of *C. lavandulifolium* (PRJNA681093) [12] and *Mikania micrantha* (PRJNA528368) [40] were downloaded from NCBI (https://www.ncbi.nlm.nih.gov/). *Helianthus annuus* (PRJNA345532) [41], *Lactuca sativa* (PRJNA173551) [42] were downloaded from EnsemblPlants (http://plants.ensembl.org/index.html). *C. nankingense* [43] was downloaded from the Chrysanthemum Genome Database (http://www.amwayabrc.com/zh-cn/index.html). *C. makinoi* [44] was downloaded from https://www.chrysanthemumgenome.wur.nl/. *C*. × *morifolium* (PRJNA796762) [33] was downloaded from the Figshare (https://doi.org/10.6084/m9.figshare.21655364.v2) and *C. seticuspe* (Gojo-0 v1) [45] was downloaded from the Plant Garden database (https://plantgarden.jp/en/list/t1111766/genome/t1111766.G002).

NAM/CUC members in six Asteraceae plants were identified in TBtools v1.098765 by BLAST (E-value cut-off of 1e^−5^) [46]. The AtNAC sequences were used as reference sequences. Sequence alignment of the obtained sequences was performed using MUSCLE [47]. The trimAI (−automated1 option) [48] was used to trim poorly aligned sequences and reserve reliable comparison results. The best-fit model (JTT + I + G4) was selected according to the BIC and the maximum likelihood (ML) phylogenetic tree was constructed in IQtree v2 (bootstrap replicates = 1000) [49]. The final phylogenetic tree was embellished in iTOL (https://itol.embl.de/).

### Subcellular localization

The CDS of ClNAM, which has had the termination codon removed was cloned into the binary vector pBI121-GFP. The recombinant vectors were transformed into *Agrobacterium* GV3101 and injected into *Nicotiana benthamiana* leaves. The treated tobacco was incubated for 48 h (24 h dark/24 h light) and the subcellular localization of fluorescent proteins was observed under a laser confocal microscope (SP8-WLL, Leica Microsystems, Wetzlar, Germany).

### *In situ* hybridization

The floral organs were collected from different developmental stages as previously described [10]. The desired target fragment is amplified in genomic DNA using *ClNAM* gene-specific primers (F: 5′-GGGTTTCGGTTTCATCCAAG-3′ and R: 5′-GGTAGGATACTTCTTATCCCTCACA-3′, 198 bp). After sequencing to obtain the reverse inserted plasmid, probes were constructed using the Roche DIG RNA Labeling Kit (SP6/T7). Detection time was 16 h.

### RNA extraction and qRT-PCR analysis

All samples were sampled as previously described [10]. The morphology of the sampled material is shown in Fig. S8 (see online supplementary material). The total RNA was extracted from the collected plant materials using the Plant RNA Rapid Extraction Kit (HUAYUEYANG Biotechnology, Beijing, China) and treated with RNase-free DNaseI to digest DNA. The first-strand cDNA was synthesized using Transcriptor First Strand cDNA Synthesis Kit (TaKaRa, Dalian, China) and miRNA 1st Strand cDNA Synthesis Kit (by tailing A) (Novozymes, Nanjing, China) with 1 μg of total RNA.

Gene and cla-miR164 expression analysis was performed by real-time quantitative reverse transcription-PCR (qRT-PCR), which was performed using a qTOWER3 (Analytik Jena AG，Germany). SYBR Premix Ex Taq (Takara Bio Inc., Shiga, Japan) and miRNA Universal SYBR gPCR Master Mix (Novozymes, Nanjing, China) were used for qRT-PCR in gene and cla-miR164, respectively. Three biological replicates were used to confirm the reliability of the results. *ClSAND* and *ClU6* were used as an internal control gene for qRT-PCR. The primers for qRT-PCR are shown in Table S2 (see online supplementary material). The 2^-ΔΔCt^ method was used to analyse qRT-PCR data.

### Transcriptome sequencing

These materials were taken from the S6 reproductive buds of the top of the wild-type and OE-*ClNAM* lines of *C. lavandulifolium*. A total of six libraries (WT-1, WT-2, WT-3, OE8–1, OE8–2, OE8–3) were constructed for RNA-seq. In conclusion, total RNA was extracted from the samples and the cDNA libraries were sequenced on the Illumina NovaSeq 6000 sequencing platform (Illumina, San Diego, CA, USA). Sequencing was performed by Biomarker Technologies (Beijing, China). The data were processed as previously described [12].

### Transcriptional activation assay

The coding regions of *ClNAM*, *ClLBD18*, *ClAP1*, and *ClRCD2*/*7* were cloned into the pGADT7 (AD) vector. The promoters of *ClNAM*, *ClCUC2a*/*c*, *ClAP1*, and *ClAG1* were isolated from *C. lavandulifolium* gDNA and cloned into the pAbAi vector. The Yeast single hybridization (Y1H) assay used the Matchmaker™ Gold Yeast Two-Hybrid System (Clontech, Mountain View, CA, USA). The yeast strain is Y1H Gold. The blank pGADT7(AD) vector was used as a negative control for this assay.

The coding regions of *ClNAM*, *ClLBD18*, *ClAP1*, and *ClRCD2*/*7* were cloned into pGreenII 62-SK. The promoters of *ClNAM*, *ClCUC2a*/*c*, *ClAP1*, and *ClAG1* were cloned into pGreenII 0800-LUC. The blank pGreenII 62-SK vector was used as a negative control for this assay. The recombinant vectors were transformed into *Agrobacterium* GV3101 and injected into *N. benthamiana* leaves. The treated tobacco was incubated for 48 h (24 h dark/24 h light). The Dual-Luciferase Repoter Assay System (Promega, Madison, WI, USA) and the multifunctional enzyme labeler EnVision (PerkinElmer, USA) were used for sample extraction and signal detection. Dual luciferase imaging was accomplished using the molecular imaging system LB983 NightOwl II (Berthold Technologies, Bad Wildbad, Germany).

### Protein interaction assay

The coding sequences of all genes to be tested were cloned into the pGADT7 (AD) and pGBKT7 (BD) vectors, respectively, for Yeast two hybrid (Y2H) assay. The experimental procedure was performed as previously described [26].

The coding sequences of all genes to be tested were cloned into pCAMBIA1300-cLUC and pCAMBIA1300-nLUC vectors, respectively (requiring the removal of the termination codon if they cloned into pCAMBIA1300-nLUC), and luciferase complementation assay was performed. The experimental procedure was as previously described [26]. Using the same method, the gene to be tested was cloned into the pSPYCE and pSPYNE173. The positive control plasmids and experimental procedure of BiFC were in reference to Lu *et al.* [50].

### Cla-miR164-guided cleavage assay

The precursor sequence of cla-miR164 was isolated using *C. lavandulifolium* genomic information [12] and cloned into pGreenII 62-SK. The 35S promoter fragment from the pBI121 vector was ligated to pGreenII 0800-LUC, designated GreenII 0800-35S::LUC. The cla-miR164 targeting site sequence in *ClNAM* was cloned into GreenII 0800-35S::LUC. In addition, the sequence was point mutated at two sites and also cloned into GreenII 0800-35S::LUC. The recombinant vectors were transformed into *Agrobacterium* GV3101 and injected into *N. benthamiana* leaves. The treated tobacco was incubated for 48 h (24 h dark/24 h light) and detected using the molecular imaging system LB983 NightOwl II (Berthold Technologies, Bad Wildbad, Germany).

### Statistical analysis

All data were analysed using IBM SPSS Statistics 23.0 (SPSS Inc.; Chicago, IL, USA) for one-way analysis of variance followed by Duncan’s test. The values are represented as the means ± SD. *P* < 0.05 was considered as statistically significant.

## Supplementary Material

Web_Material_uhae039

## Date availability

Raw sequence data were submitted to the Sequence Read Archive (SRA) database of the National Center for Biotechnology Information (NCBI) (accession number: PRJNA956717).
