# Supplementary material for: cla-miR164-NO APICAL MERISTEM (ClNAM) regulates the inflorescence architecture development of Chrysanthemum lavandulifolium
Source: Hortic Res. 2024 Feb 22;11(4):uhae039. doi: 10.1093/hr/uhae039 (PMC11017518; doi:10.1093/hr/uhae039)
Supplement: Web_Material_uhae039 [file web_material_uhae039.zip › Supplementary Figure.pdf]

A

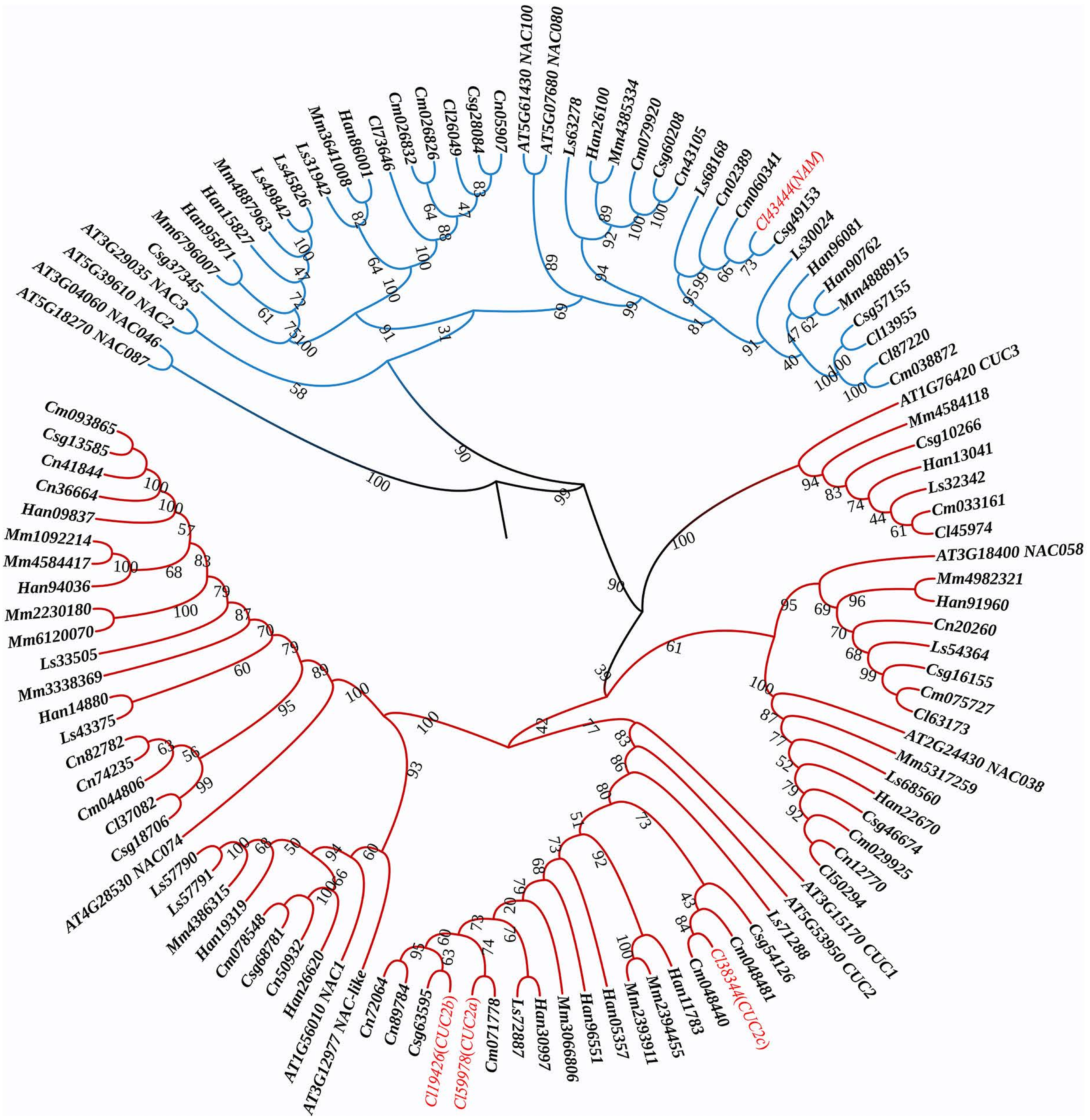

B

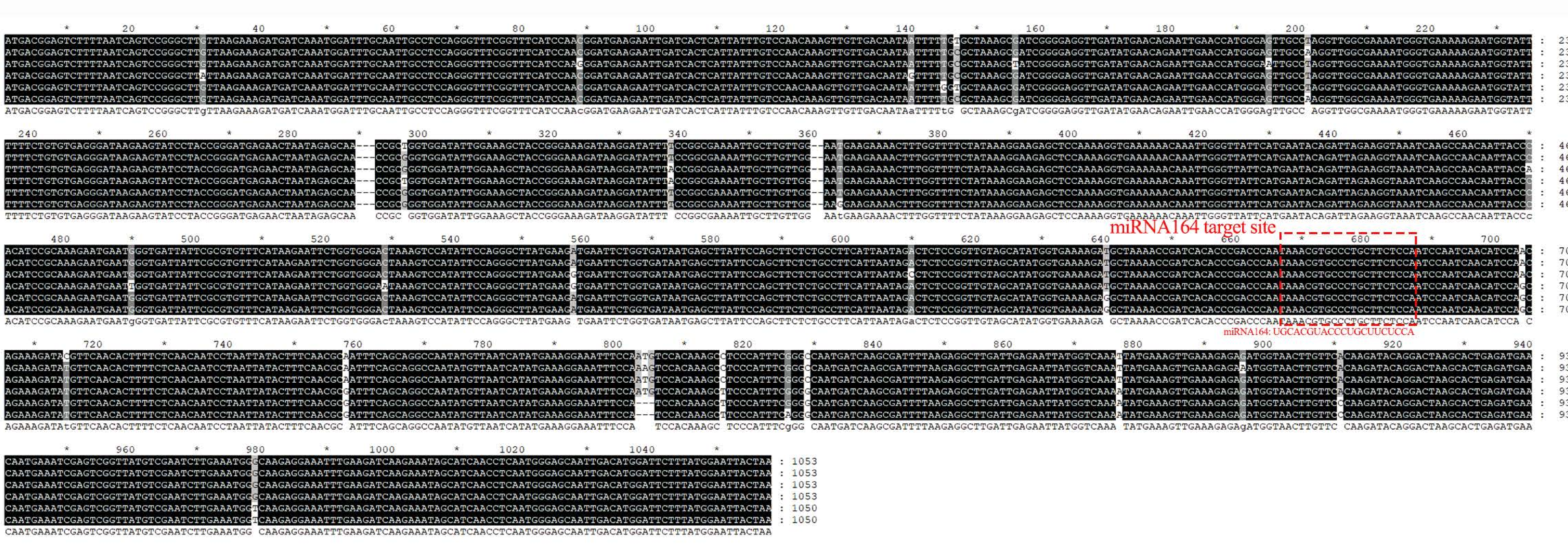

Supplemental Figure 1 Phylogenetic analysis and sequences conservatism of *ClNAM* in Chrysanthemum. *ClNAM* was the ortholous gene of AtNAC080 and AtNAC100. All ortholous genes of ClNAM in Chrysanthemum were have conservative miRNA164 target site.

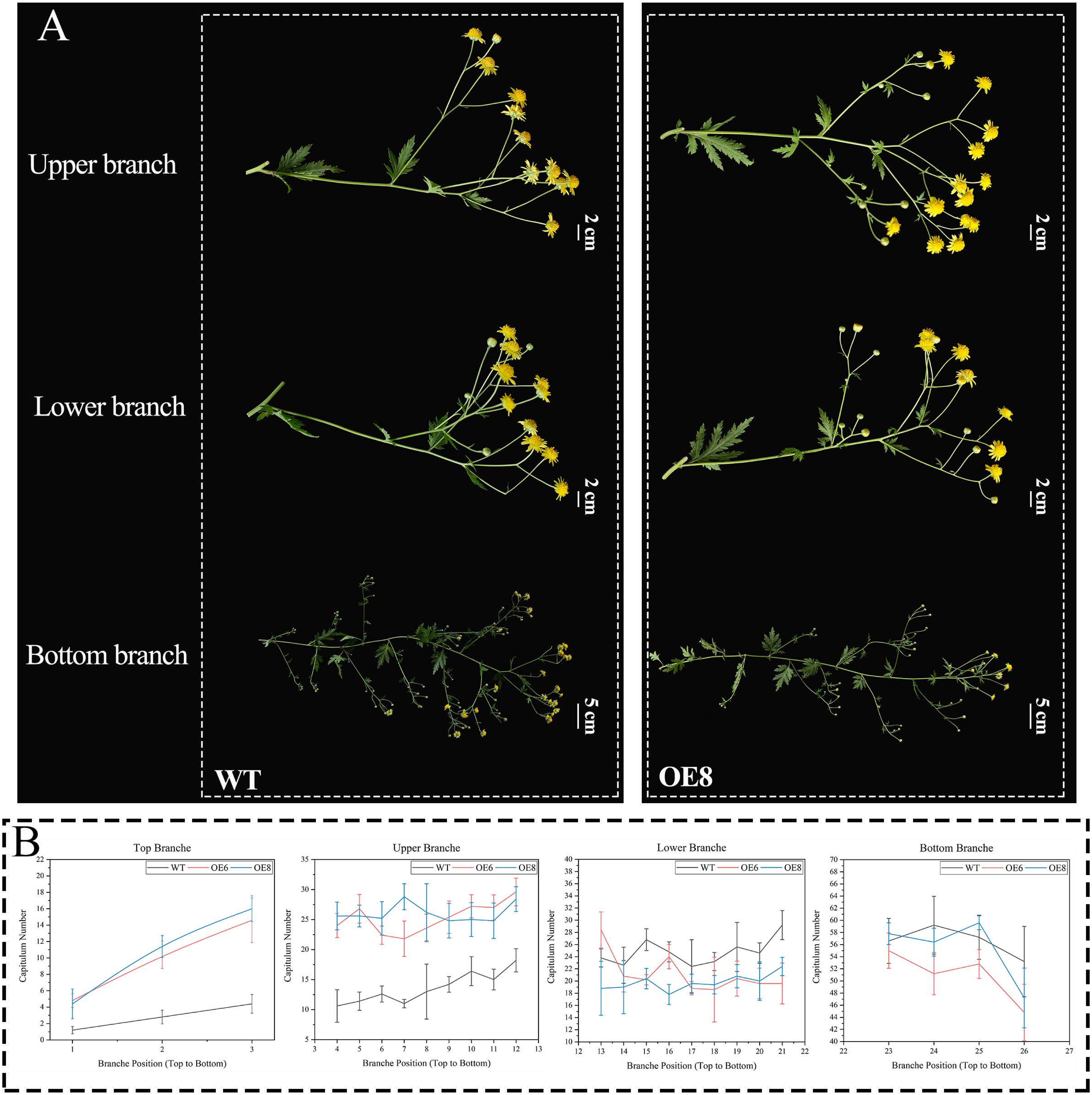

Supplemental Figure 2 Overexpression of *CINAM* effacted the number of capitulum on upper, lower, and bottom branch.

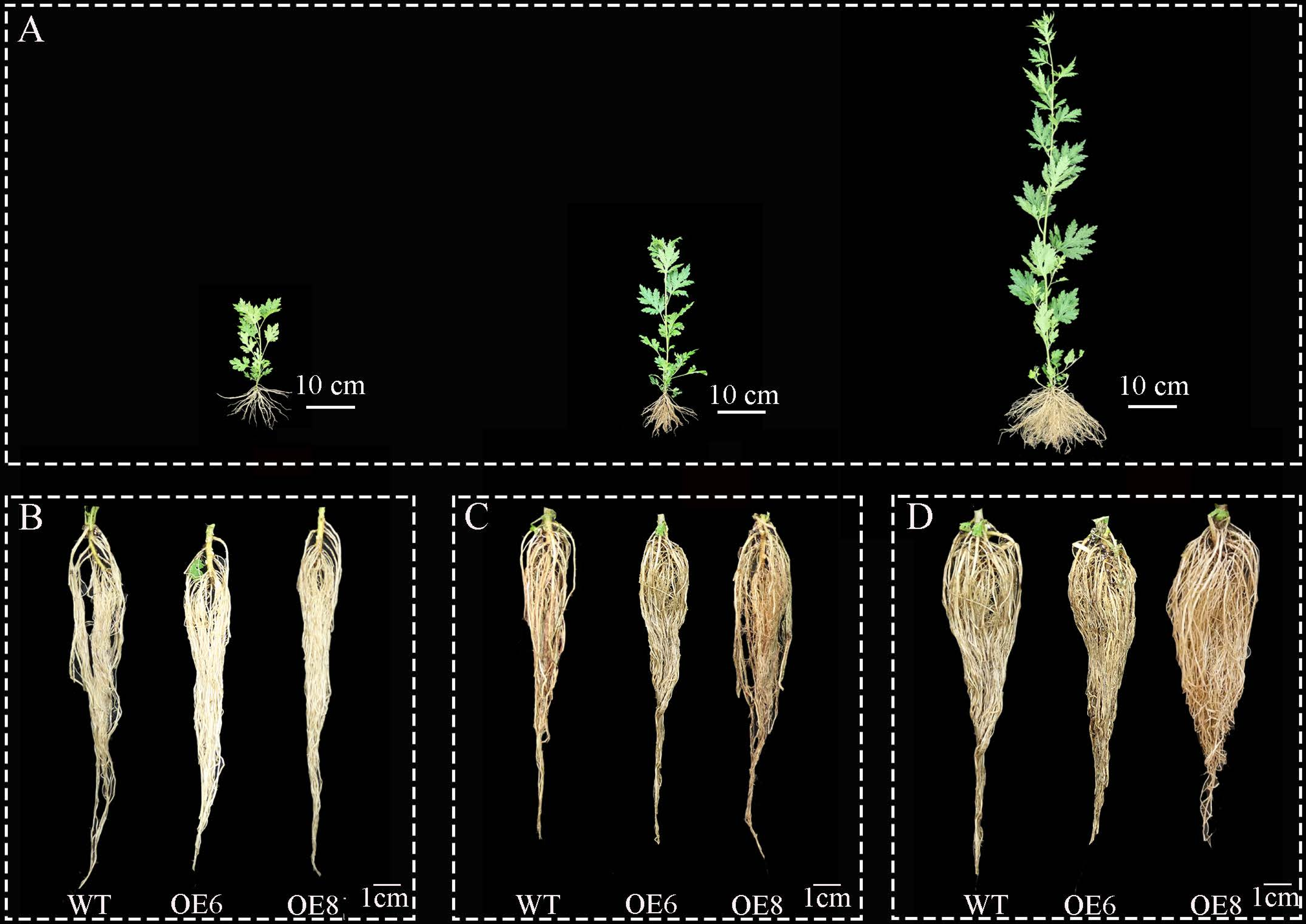

Supplemental Figure 3 Overexpression of *ClNAM* did not affect the development of *C. lavandulifolium* roots.

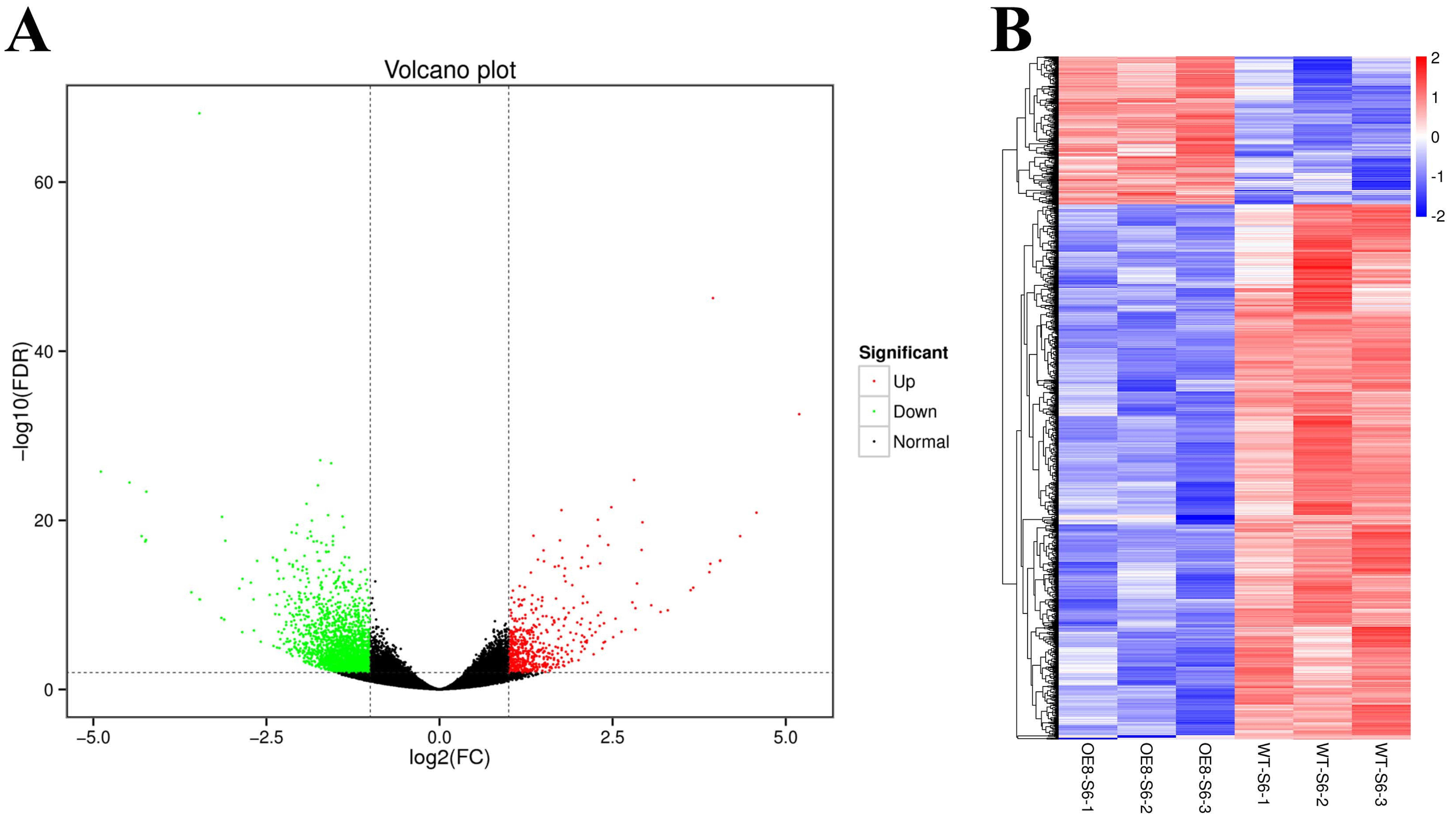

Supplemental Figure 4 Differentially expressed genes bettwen WT and OE-*ClNAM* lines.

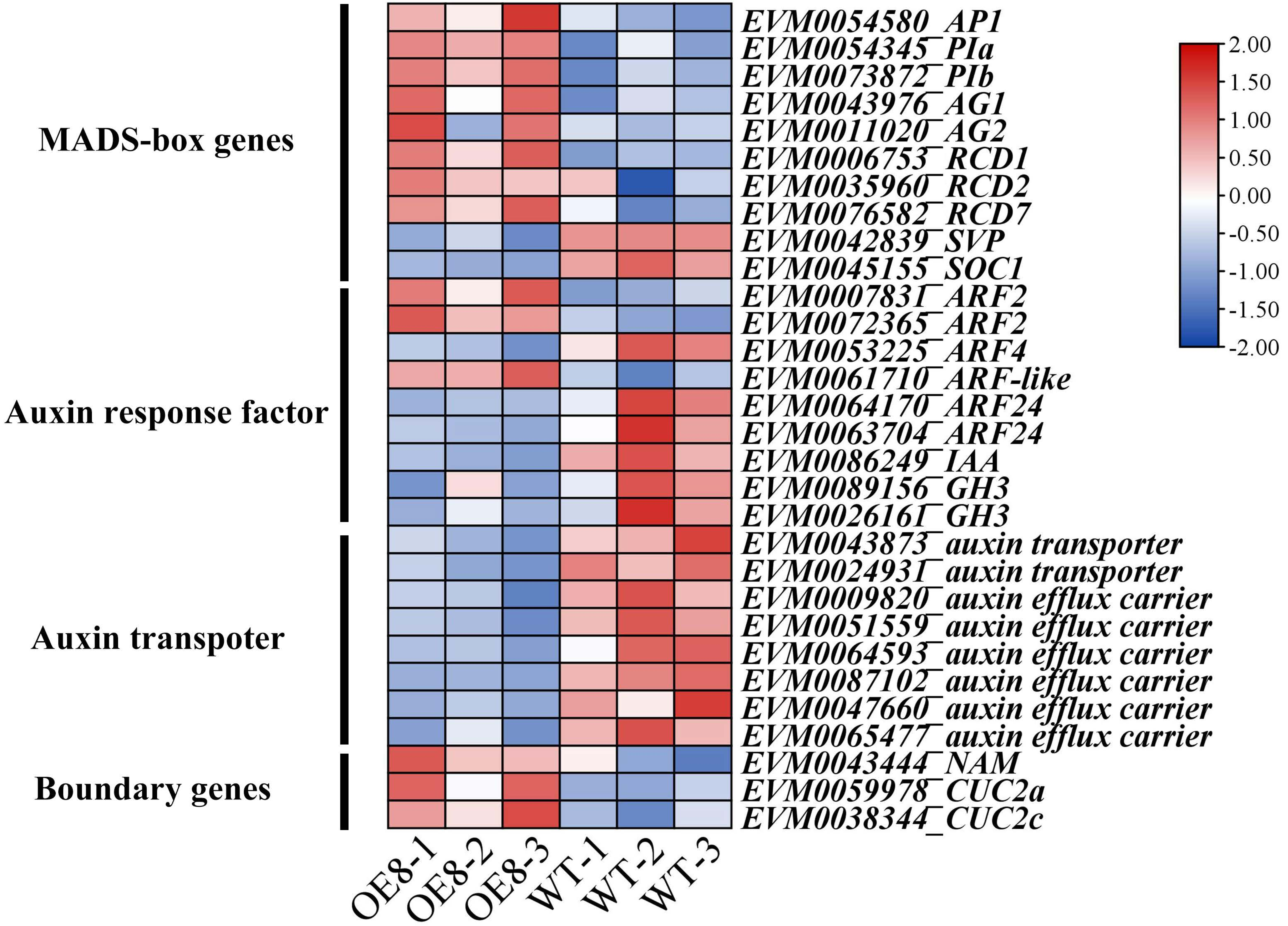

Supplemental Figure 5 Heat map of genes associated with flower development that are differentially expressed in the transcriptome

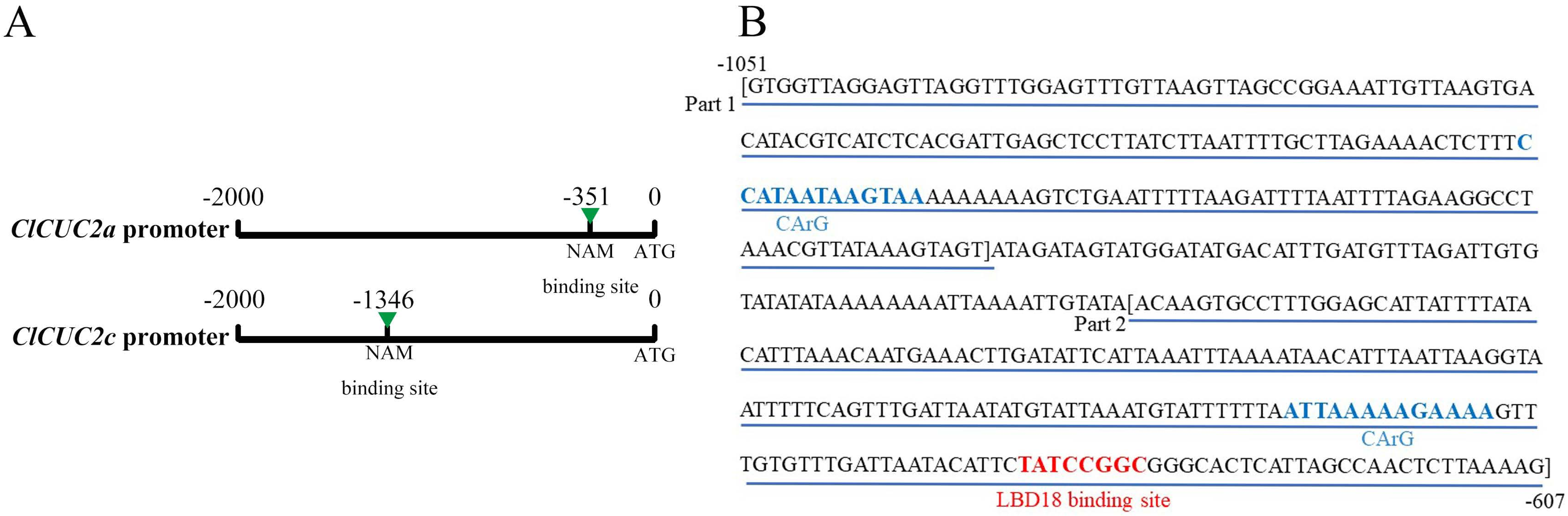

Supplemental Figure 6 Sequence analysis of *ClCUC2a/c* and *ClNAM* promoter.

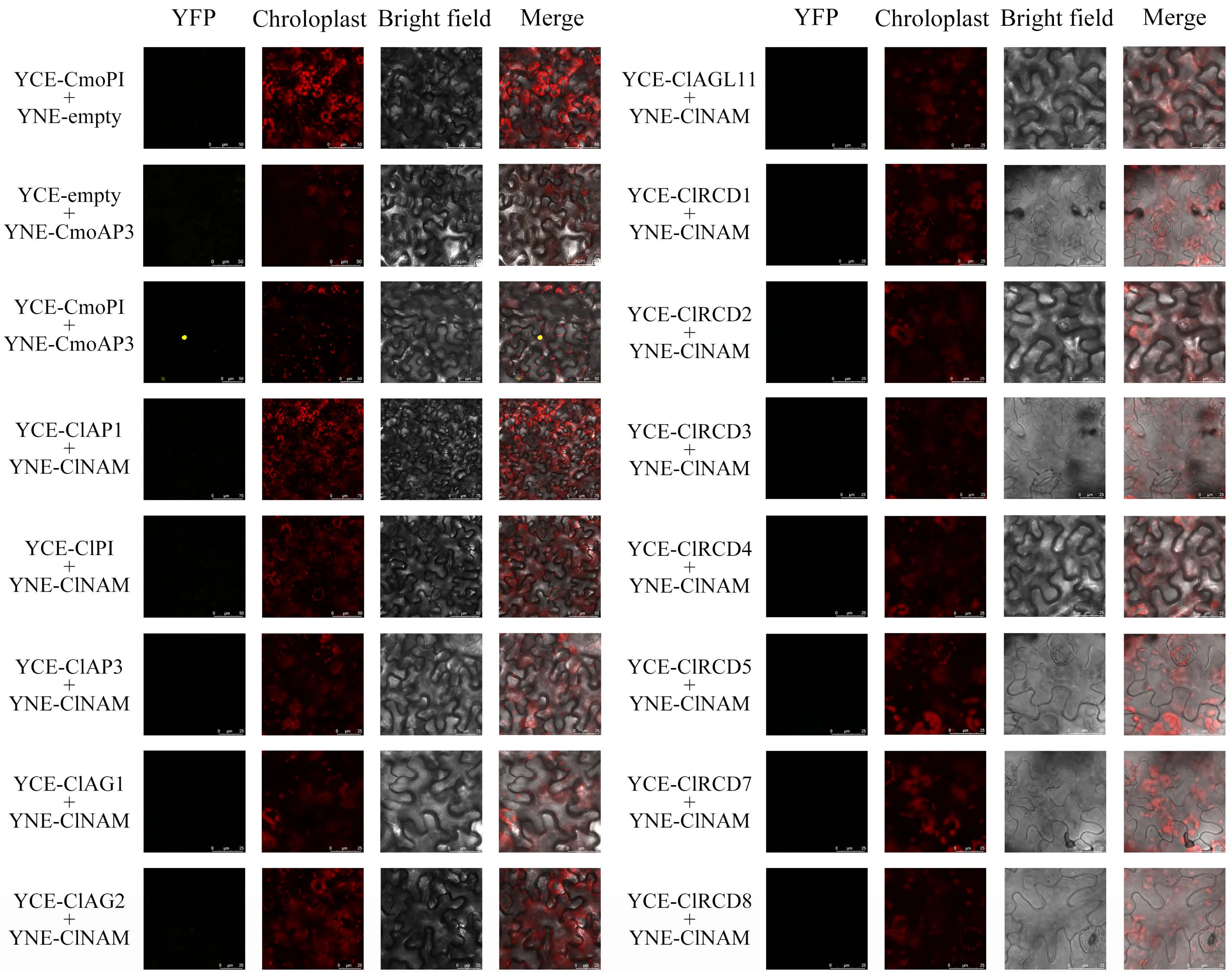

Supplemental Figure 7 Interaction of ClNAM with ABC(D)E-class MADS-box proteins was not observed in the BiFC results.

Positive results of CmoAP3-CmoPI were used as positive control in this experiment. (Cmo: *C.* × *morifolium*, Lu et al., 2022).

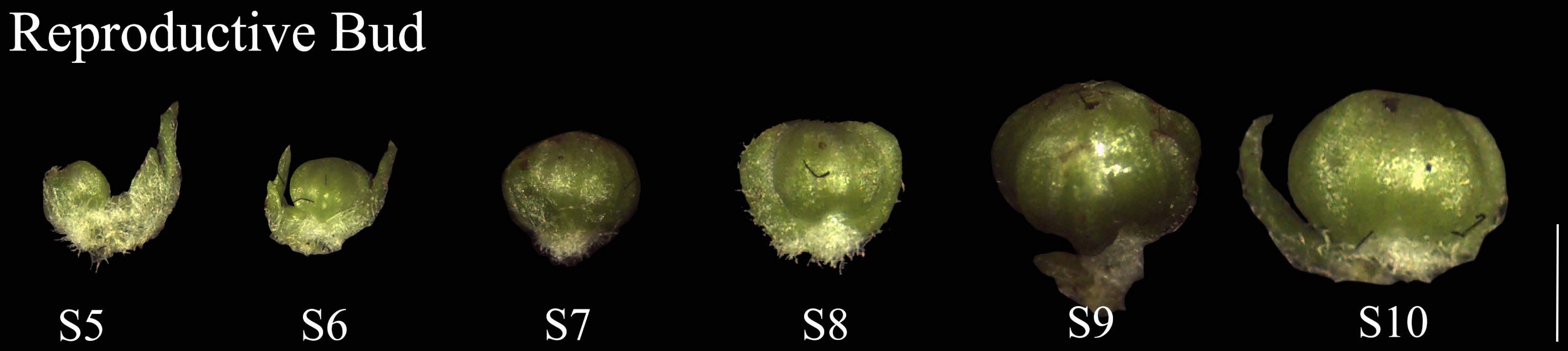

Supplemental Figure 7 The morphology of the sampled material. bar = 1 mm
